# Supplementary material for: A systematic review of the clinical profile of patients with bubonic plague and the outcome measures used in research settings
Source: PLoS Negl Trop Dis. 2023 Nov 9;17(11):e0011509. doi: 10.1371/journal.pntd.0011509 (PMC10662759; doi:10.1371/journal.pntd.0011509)
Supplement: S1 Text — (DOCX) [file pntd.0011509.s001.docx]

All searches were conducted in September 2021

**PubMED**

| *Search terms:* ((("Bubonic plague"[Mesh]) OR (((plague[Title/Abstract]) OR "pasturella pestis"[Title/Abstract]) OR "yersinia pestis"[Title/Abstract]))) NOT (((("Animals"[Mesh] NOT ("Animals"[Mesh] AND "Humans"[Mesh])))))  *Article type*: case reports; clinical study; clinical trial; comparative study; controlled clinical trial; multicenter study; observational study; pragmatic clinical trial; randomised controlled trial  *Species*: humans |
| --- |

**JSTOR**

| *Search terms*: plague OR "pasteurella pestis" OR "Yersinia pestis"  *Item type*: articles  *Search within results*: (patient* OR case* OR participant* OR subject* OR infant* OR child* OR adult*) |
| --- |

**Cochrane CENTRAL**

| *Search terms*: ((("Plague"[Mesh]) OR (((plague[Title/Abstract]) OR "pasteurella pestis"[Title/Abstract]) OR "yersinia pestis"[Title/Abstract]))) NOT (((("Animals"[Mesh] NOT ("Animals"[Mesh] AND "Humans"[Mesh]))))) |
| --- |

**International Clinical Trial Registry Platform (ICTRP)**

| *Search term*: plague |
| --- |

**ISRCTN**

| *Search term*: plague |
| --- |

**Clinicaltrials.gov**

| *Search term*: plague |
| --- |
