# Supplementary material for: A systematic review of the clinical profile of patients with bubonic plague and the outcome measures used in research settings
Source: PLoS Negl Trop Dis. 2023 Nov 9;17(11):e0011509. doi: 10.1371/journal.pntd.0011509 (PMC10662759; doi:10.1371/journal.pntd.0011509)
Supplement: S2 Table — (DOCX) [file pntd.0011509.s003.docx]

**S2a – Risk of bias assessment for case reports**

| **Study title**  ***First author, Year*** | **Demographic characteristics** | **Patient history** | **Current clinical condition** | **Diagnostic tests/assessment** | **Intervention/ treatment** | **Post-intervention clinical condition** | **Adverse/ unanticipated events** | **Takeaway lessons** |
| --- | --- | --- | --- | --- | --- | --- | --- | --- |
| Two Cases Of Bubonic Plague Occurring On Board Ship  *Barnett H. N., 1902 [1]* | No | No | Yes | No | No | No | Unclear | Yes |
| A case of bubonic plague on a vessel arriving in the Mersey  *NK, 1905 [2]* | No | No | Yes | No | N/A | N/A | N/A | Yes |
| Subacute plague in man due to ground squirrel infection  *McCoy G. W., 1909 [3]* | Yes | Yes | Yes | Yes | No | Yes | Yes | Yes |
| Three Cases of Bubonic Plague Arising In England  *Rendle-Short A., 1916 [4]* | No | No | Yes | No | No | No | No | Yes |
| Plague -- New Mexico  *United States Centres for Disease Control and Prevention, 1965 [5]* | No | Yes | Yes | Yes | No | Yes | Yes | Yes |
| Plague: Shasta County, California  *United States Centres for Disease Control and Prevention, 1965 [6]* | No | Yes | Yes | Yes | No | Unclear | N/A | Yes |
| Suspected Case of Imported Bubonic Plague  *United States Centres for Disease Control and Prevention, 1966 [7]* | No | Yes | No | Yes | No | Unclear | N/A | Yes |
| Plague -- Arizona  *United States Centres for Disease Control and Prevention, 1967 [8]* | No | Yes | Yes | Yes | No | Unclear | N/A | Yes |
| Plague in San Diego  *Connor J. D., 1968 [9]* | Yes | Yes | Yes | Yes | No | N/A | N/A | Yes |
| Presumptive Bubonic Plague — Denver, Colorado  *United States Centres for Disease Control and Prevention, 1968 [10]* | No | Yes | Yes | Yes | No | Yes | Yes | Yes |
| Bubonic Plague Death -- Lemhi County, Idaho  *United States Centres for Disease Control and Prevention, 1968 [11]* | No | No | Yes | Yes | No | No | N/A | Yes |
| Plague Case -- Navajo Reservation -- Kayenta, Arizona  *United States Centres for Disease Control and Prevention, 1968 [12]* | No | Yes | Yes | Yes | No | No | Unclear | No |
| Plague -- New Mexico  *United States Centres for Disease Control and Prevention, 1969 [13]* | No | Yes | Yes | Yes | No | No | Unclear | No |
| Bubonic plague in the Southwestern United States  *Reed W. P., 1970 [14]* | No | Yes | Yes | Yes | No | Yes | Yes | Yes |
| Bubonic Plague – California  *United States Centres for Disease Control and Prevention, 1970 [15]* | No | Unclear | Unclear | Yes | No | No | N/A | No |
| Bubonic Plague – California  *United States Centres for Disease Control and Prevention, 1970* | No | Yes | Yes | Yes | No | No | N/A | Yes |
| Human Bubonic Plague -- Cochiti, New Mexico  *United States Centres for Disease Control and Prevention, 1970 [16]* | No | Yes | Yes | Yes | Yes | Yes | N/A | Yes |
| Bubonic Plague -- Santa Fe, New Mexico  *United States Centres for Disease Control and Prevention, 1970 [17]* | No | N/A | Yes | Yes | No | Unclear | N/A | Yes |
| Plague -- New Mexico  *United States Centres for Disease Control and Prevention, 1970 [18]* | No | Yes | Yes | Yes | No | Yes | N/A | Yes |
| Plague -- New Mexico  *United States Centres for Disease Control and Prevention, 1970 [19]* | No | Yes | Yes | Yes | No | No | N/A | Yes |
| Plague -- California  *United States Centres for Disease Control and Prevention, 1970 [20]* | No | Yes | Yes | Yes | No | No | N/A | Yes |
| Plague - Rio en Medio, New Mexico  *United States Centres for Disease Control and Prevention, 1970 [21]* | No | Yes | Yes | Yes | No | Unclear | N/A | Yes |
| Plague – New Mexico  *United States Centres for Disease Control and Prevention, 1970 [22]* | No | Yes | Yes | Yes | No | No | Unclear | No |
| Human Bubonic Plague – Oregon  *United States Centres for Disease Control and Prevention, 1971 [23]* | No | Yes | Yes | Yes | No | No | Unclear | No |
| Human Bubonic Plague – New Mexico  *United States Centres for Disease Control and Prevention, 1971 [24]* | No | Yes | Yes | Yes | No | Yes | N/A | Yes |
| Human Bubonic Plague - Coconino County Colorado  *United States Centres for Disease Control and Prevention, 1972 [25]* | No | Yes | Yes | Yes | No | No | N/A | Yes |
| Human Bubonic Plague – New Mexico  *United States Centres for Disease Control and Prevention, 1974 [26]* | No | Yes | Yes | No | N/A | N/A | N/A | Yes |
| Human Plague – New Mexico  *United States Centres for Disease Control and Prevention, 1974 [27]* | No | Yes | Yes | Yes | No | No | N/A | Yes |
| Human Plague – New Mexico, Utah  *United States Centres for Disease Control and Prevention, 1974 [28]* | No | Yes | Yes | Yes | No | No | N/A | Yes |
| Plague and the gallium scan  *Stahly T. L., 1975 [29]* | No | Yes | Yes | Yes | No | Yes | Yes | Yes |
| Fatal Human Plague – California  *United States Centres for Disease Control and Prevention, 1975 [30]* | No | Yes | Yes | Yes | No | Yes | N/A | Yes |
| Bubonic Plague – Arizona  *United States Centres for Disease Control and Prevention, 1975 [31]* | No | No | Yes | No | No | No | Unclear | Yes |
| Plague in Humans – New Mexico  *United States Centres for Disease Control and Prevention, 1975 [32]* | No | No | No | Yes | No | No | No | N/A |
| Human Plague Case -- Bernalillo County, New Mexico  *United States Centres for Disease Control and Prevention, 1975 [33]* | No | Yes | Yes | Yes | Yes | Yes | Yes | Yes |
| Bubonic Plague from Exposure to a Rabbit: A Documented Case, and a Review of Rabbit-Associated Plague Cases in The United States  *Von Reyn C. F., 1976 [34]* | No | Yes | Yes | Yes | No | Yes | N/A | Yes |
| Human Plague -- Arizona, California, New Mexico  *United States Centres for Disease Control and Prevention, 1976 [35]* | No | Yes | Yes | Yes | No | Unclear | No | Yes |
| Plague and pregnancy. A case report  *Mann J, 1977 [36]* | Yes | Yes | Yes | Yes | Yes | Yes | N/A | Yes |
| Plague -- Arizona, Colorado, New Mexico  *United States Centres for Disease Control and Prevention, 1977 [37]* | No | Yes | Yes | Yes | No | No | Yes | Yes |
| Plague – United States  *United States Centres for Disease Control and Prevention, 1977 [38]* | No | Yes | Yes | Yes | No | Unclear | N/A | Yes |
| Plague -- Arizona, California, New Mexico  *United States Centres for Disease Control and Prevention, 1978 [39]* | No | Yes | Yes | Yes | No | Yes | N/A | Yes |
| Plague in the United States: the "black death" is still alive  *Hoffman S. L., 1980 [40]* | No | Yes | Yes | Yes | No | Yes | N/A | Yes |
| Plague -- United States  *United States Centres for Disease Control and Prevention, 1980 [41]* | No | Yes | Yes | Yes | Yes | Yes | N/A | Yes |
| Human Plague – Texas, New Mexico  *United States Centres for Disease Control and Prevention, 1981 [42]* | No | Yes | Yes | Yes | No | Yes | Yes | Yes |
| Human plague associated with domestic cats--California, Colorado  *United States Centres for Disease Control and Prevention, 1981 [43]* | No | Yes | Yes | Yes | Yes | Yes | Yes | Yes |
| Peripatetic Plague  *Mann J., 1982 [44]* | No | Yes | Yes | Yes | Yes | Yes | N/A | Yes |
| Plague - South Carolina  *United States Centres for Disease Control and Prevention, 1983 [45]* | No | Yes | Yes | Yes | No | Yes | No | Yes |
| Plague Pneumonia – California  *United States Centres for Disease Control and Prevention, 1984 [46]* | No | Yes | No | Yes | No | No | No | Yes |
| Winter Plague -- Colorado, Washington, Texas, 1983-1984  *United States Centres for Disease Control and Prevention, 1984 [47]* | No | Yes | No | Yes | No | No | No | Yes |
| Human Bubonic Plague Transmitted by a Domestic Cat Scratch  *Weniger B. G., 1984 [48]* | No | Yes | Yes | Yes | No | Yes | N/A | Yes |
| Nineteen cases of plague in Arizona. A spectrum including ecthyma gangrenosum due to plague and plague in pregnancy  *Welty T. K., 1985 [49]* | No | Yes | Yes | Yes | No | Yes | Yes | Yes |
| Multiple lung cavities in a 12-year-old girl with bubonic plague, sepsis, and secondary pneumonia  *Florman A. L., 1986 [50]* | Yes | Yes | Yes | Yes | Yes | Yes | N/A | Yes |
| Plague in a pregnant patient  *Wong T. W., 1986 [51]* | No | Yes | Yes | Yes | Yes | Yes | N/A | Yes |
| Imaging in plague  *Moreno A. J., 1987 [52]* | No | No | Yes | Yes | No | Yes | Yes | Yes |
| Human Plague -- United States, 1988  *United States Centres for Disease Control and Prevention, 1988 [53]* | No | No | No | Unclear | No | No | No | No |
| Imported bubonic plague -- District of Columbia  *United States Centres for Disease Control and Prevention, 1990 [54]* | No | Yes | Yes | Yes | Yes | Yes | N/A | No |
| Bubonic plague in a child presenting with fever and altered mental status  *Migden D., 1990 [55]* | No | Yes | Yes | Yes | No | Yes | N/A | Yes |
| Plague in New Mexico  *Owens C., 1990 [56]* | No | Yes | Yes | Yes | No | No | N/A | Yes |
| An Outbreak of Plague in Northwestern Province, Zambia  *McClean K. L., 1995 [57]* | Yes | Yes | Yes | Yes | No | Yes | Yes | Yes |
| Fatal human plague--Arizona and Colorado, 1996  *United States Centres for Disease Control and Prevention, 1997 [58]* | No | Yes | Yes | Yes | N/A | N/A | N/A | Yes |
| Cases of cat-associated human plague in the Western US, 1977-1998  *Gage K. L., 2000 [59]* | No | Yes | Yes | Yes | No | No | Yes | Yes |
| Imported plague--New York City, 2002  *United States Centres for Disease Control and Prevention, 2003 [60]* | No | Yes | Yes | Yes | No | Yes | Yes | Yes |
| Painful lymphadenopathy and fulminant sepsis in a previously healthy 16-year-old girl  *Chmura K., 2003 [61]* | No | Yes | Yes | Yes | N/A | N/A | N/A | Yes |
| Human plague--four states, 2006  *United States Centres for Disease Control and Prevention, 2006 [62]* | No | Yes | No | Yes | No | No | No | Yes |
| Notes from the field: two cases of human plague--Oregon, 2010  *United States Centres for Disease Control and Prevention, 2011 [63]* | No | No | No | No | No | No | N/A | Yes |
| Misidentification of Yersinia pestis by Automated Systems, Resulting in Delayed Diagnoses of Human Plague Infections—Oregon and New Mexico, 2010–2011  *Tourdjman M., 2012 [64]* | No | Yes | Yes | Yes | No | Yes | Yes | Yes |
| Case report  *Lazet K., 2018 [65]* | No | Yes | Yes | Yes | Yes | Yes | N/A | Yes |
| Human case of bubonic plague resulting from the bite of a wild Gunnison’s prairie dog during translocation from a plague endemic area  *Melman S. D., 2018 [66]* | No | Yes | Yes | Yes | No | Yes | N/A | Yes |
| Two fatal cases of plague after consumption of raw marmot organs  *Kehrmann J., 2020 [67]* | Yes | Yes | Yes | Yes | Yes | Yes | Yes | Yes |
| Delays in Identification and Treatment of a Case of Septicemic Plague — Navajo County, Arizona, 2020  *Dale A. P., 2021 [68]* | Yes | Yes | Yes | Yes | Yes | Yes | NA | Yes |

**S2b – Risk of bias assessment for quasi-experimental studies**

| **Study title**  ***First author, Year*** | **Clear cause and effect** | **Participants similar in comparisons** | **Participants receiving similar treatment** | **Control group** | **Multiple measurements of outcome** | **Follow-up complete** | **Outcomes measured in same way** | **Outcomes measured in reliable way** | **Appropriate statistical analysis used** |
| --- | --- | --- | --- | --- | --- | --- | --- | --- | --- |
| Streptomycin in Bubonic Plague  *Haddad C. H., 1948 [69]* | No | Unclear | Unclear | No | No | Unclear | Unclear | Unclear | N/A |
| Co-trimoxazole in Bubonic Plague  *Nguyen-Van-Ai, 1973 [70]* | No | N/A | N/A | No | Unclear | Unclear | N/A | Unclear | N/A |
| Yersinia pestis Infection in Vietnam. II; Quantitative Blood Cultures and Detection of Endotoxin in the Cerebrospinal Fluid of Patients with Meningitis  *Butler T, 1976 [71]* | Yes | No | Yes | No | No | Unclear | Yes | Yes | N/A |

**S2c – Risk of bias assessment for cohort studies**

| **Study title**  ***First author, Year*** | **Similarities between groups** | **Similarity of measurement exposures** | **Exposure measured reliably** | **Confounding factors identified** | **Strategies to manage confounders** | **Groups free of outcome at moment of exposure** | **Measurement of outcomes reliable** | **Sufficient follow-up time** | **Follow-up complete** | **Strategies to address incomplete follow-up** | **Appropriate statistical analysis** |
| --- | --- | --- | --- | --- | --- | --- | --- | --- | --- | --- | --- |
| Clinical Features of Plague in the United States: the 1969-1970 Epidemic  *Palmer D. L., 1971 [72]* | N/A | N/A | Yes | No | N/A | Unclear | Unclear | No | Unclear | No | N/A |
| Yersinia pestis Infection in Vietnam. I. Clinical and Hematologic Aspects  *Butler T., 1974 [73]* | N/A | N/A | Yes | No | N/A | Unclear | Yes | Yes | Unclear | N/A | Yes |
| Epidemiological and clinical features of an outbreak of bubonic plague in New Mexico  *Von Reyn C. F., 1977 [74]* | N/A | N/A | Yes | No | No | Unclear | Unclear | Unclear | Unclear | No | Yes |
| Plague in the United States 1982  *Barnes A. M., 1983 [75]* | N/A | N/A | Yes | No | N/A | Unclear | Yes | Yes | Yes | N/A | Yes |
| Plague meningitis--a retrospective analysis of cases reported in the United States, 1970-1979  *Becker T. M., 1987 [76]* | Unclear | Yes | Yes | No | N/A | N/A | Yes | N/A | N/A | N/A | Yes |
| Plague - A clinical review of 27 cases  *Crook L. D., 1992 [77]* | N/A | No | Yes | No | No | Unclear | Yes | No | Unclear | No | N/A |
| Current epidemiology of human plague in Madagascar  *Chanteau S., 2000 [78]* | Unclear | N/A | N/A | No | N/A | No | Yes | N/A | N/A | N/A | Yes |
| Gentamicin and Tetracyclines for the Treatment of Human Plague: Review of 75 cases in New Mexico, 1985-1999  *Boulanger L. L., 2004 [79]* | N/A | N/A | Yes | No | N/A | No | Yes | Unclear | Unclear | No | Yes |
| Plague Outbreak in Libya, 2009, Unrelated to Plague in Algeria  *Cabanel N., 2013 [80]* | N/A | Yes | Yes | Yes | No | Yes | Yes | Yes | Yes | N/A | Yes |
| Outbreak of Plague in a High Malaria Endemic Region — Nyimba District, Zambia, March–May 2015  *Sinyange N., 2016 [81]* | N/A | N/A | Yes | No | No | Unclear | Unclear | Unclear | Unclear | Unclear | Yes |
| Successful Treatment of Human Plague with Oral Ciprofloxacin  *Apangu T., 2017 [82]* | Yes | Yes | Yes | No | N/A | No | No | Yes | Yes | N/A | N/A |

**S2d – Risk of bias assessment for randomised controlled trials**

| **Study title**  ***First author, Year*** | **True randomisation** | **Concealed allocation** | **Treatment groups similar at baseline** | **Participants blind to allocation** | **Those delivering treatment blind to allocation** | **Outcome assessors blind to allocation** | **Groups treated identically** | **Follow-up complete** | **Participants analysed in groups allocated at randomisation** | **Outcomes measured in same way** | **Outcomes measured in reliable way** | **Appropriate statistical analysis** | **Protocol deviations accounted for** |
| --- | --- | --- | --- | --- | --- | --- | --- | --- | --- | --- | --- | --- | --- |
| Treatment of Plague with Gentamicin or Doxycycline in a Randomized Clinical Trial in Tanzania  *Mwengee W., 2006 [83]* | Yes | Unclear | Yes | No | No | No | Yes | No | Yes | Yes | Yes | Yes | Yes |

References

1. Anderson TL. Two Cases of Bubonic Plague on Board Ship. British Medical Journal. 1902;2(2175):730-. doi: 10.1136/bmj.2.2175.730-b.

2. The Plague. A Case Of Bubonic Plague On A Vessel Arriving In The Mersey. The British Medical Journal. 1905;1(2303):383-4.

3. McCoy GW, Wherry WB. Subacute Plague in Man Due to Ground Squirrel Infection. The Journal of Infectious Diseases. 1909;6(5):670-5.

4. Rendle Short A. Three Cases Of Bubonic Plague Arising In England. BMJ. 1916;2(2905):1.

5. PLAGUE - New Mexico. Morbidity and Mortality. 1965;14(33):285-6.

6. PLAGUE: Shasta County, California. Morbidity and Mortality. 1965;14.

7. SUSPECTED CASE OF IMPORTED BUBONIC PLAGUE - Texas. Morbidity and Mortality. 1966;15.

8. PLAGUE — Arizona. Morbidity and Mortality. 1967;16(27):222-.

9. Connor JD, Williams RA, Thompson MA, Ginsberg M, Daley S. Plague in San Diego. West J Med. 1978;129(5):394-406. Epub 1978/11/01. PubMed PMID: 726420; PubMed Central PMCID: PMCPMC1238391.

10. PRESUMPTIVE BUBONIC PLAGUE — Denver, Colorado. Morbidity and Mortality. 1968;17(27):253-60.

11. BUBONIC PLAGUE DEATH – Lemhi County, Idaho. Morbidity and Mortality. 1968;17(44):405-6.

12. PLAGUE CASE - Navajo Reservation - Kayenta, Arizona. Morbidity and Mortality. 1968;17(29):269-70.

13. PLAGUE – New Mexico. Morbidity and Mortality. 1969;18(27):233-.

14. Reed WP, Palmer DL, Williams RCJ, Kisch AL. BUBONIC PLAGUE IN THE SOUTHWESTERN UNITED STATES: A REVIEW OF RECENT EXPERIENCE. Medicine. 1970;49(6).

15. BUBONIC PLAGUE - California. Morbidity and Mortality. 1970;19(23):221-2.

16. HUMAN BUBONIC PLAGUE - Cochiti, New Mexico. Morbidity and Mortality. 1970;19(20):197-8.

17. BUBONIC PLAGUE – Santa Fe, New Mexico. Morbidity and Mortality. 1970;19(32):313-4.

18. PLAGUE - New Mexico. Morbidity and Mortality. 1970;19(25):241-2.

19. PLAGUE — New Mexico. Morbidity and Mortality. 1970;19(37):370-.

20. PLAGUE — California. Morbidity and Mortality. 1970;19(45):437-8.

21. PLAGUE — Rio en Medio, New Mexico. Morbidity and Mortality. 1970;19(33):322-.

22. PLAGUE — New Mexico. Morbidity and Mortality. 1970;19(28):270-1.

23. HUMAN BUBONIC PLAGUE – Oregon. Morbidity and Mortality. 1971;20(34):303-4.

24. HUMAN BUBONIC PLAGUE - New Mexico. Morbidity and Mortality. 1971;20(32):283-4.

25. HUMAN BUBONIC PLAGUE - Coconino County, Arizona. Morbidity and Mortality. 1972;21(10):81-2.

26. HUMAN BUBONIC PLAGUE - New Mexico. Morbidity and Mortality. 1974;23(26):231-2.

27. HUMAN PLAGUE - New Mexico. Morbidity and Mortality. 1974;23(41):350-.

28. HUMAN PLAGUE — New Mexico, Utah. Morbidity and Mortality. 1974;23(37):317-8.

29. Stahly TL, Shoop JD. Plague and the gallium scan: Case report. J Nucl Med. 1975;16(11):1031-2. Epub 1975/11/01. PubMed PMID: 1185264.

30. FATAL BUBONIC PLAGUE - California. Morbidity and Mortality. 1975;24(24):211-.

31. BUBONIC PLAGUE – Arizona. Morbidity and Mortality. 1975;24(22):190-5.

32. PLAGUE IN HUMANS - New Mexico. Morbidity and Mortality. 1975;24(40):341-2.

33. HUMAN PLAGUE CASE – Bernalillo County, New Mexico. Morbidity and Mortality. 1975;24(10):90-5.

34. von Reyn CF, Barnes AM, Weber NS, Hodgin UG. Bubonic plague from exposure to a rabbit: a documented case, and a review of rabbit-associated plague cases in the United States. Am J Epidemiol. 1976;104(1):81-7. Epub 1976/07/01. doi: 10.1093/oxfordjournals.aje.a112276. PubMed PMID: 937344.

35. Human Plague — Arizona, California, New Mexico. Morbidity and Mortality Weekly Report. 1976;25(19):155-.

36. Mann JM, Moskowitz R. Plague and Pregnancy: A Case Report. JAMA. 1977;237(17):1854-5. doi: 10.1001/jama.1977.03270440044020.

37. Plague — Arizona, Colorado, New Mexico. Morbidity and Mortality Weekly Report. 1977;26(26):215-6.

38. Plague — United States. Morbidity and Mortality Weekly Report. 1977;26(41):337-.

39. Plague — Arizona, California, New Mexico. Morbidity and Mortality Weekly Report. 1978;27(30):259-60.

40. Hoffman SL. Plague in the United States: the "black death" is still alive. Ann Emerg Med. 1980;9(6):319-22. Epub 1980/06/01. doi: 10.1016/s0196-0644(80)80068-0. PubMed PMID: 7386958.

41. Plague — United States, 1980. Morbidity and Mortality Weekly Report. 1980;29(31):371-7.

42. Human Plague — Texas, New Mexico. Morbidity and Mortality Weekly Report. 1981;30(12):137-8.

43. Human Plague Associated with Domestic Cats — California, Colorado. Morbidity and Mortality Weekly Report. 1981;30(22):265-6.

44. Mann JM, Schmid GP, Stoesz PA, Skinner MD, Kaufmann AF. Peripatetic Plague. JAMA. 1982;247(1):47-8. doi: 10.1001/jama.1982.03320260031024.

45. Plague — South Carolina. Morbidity and Mortality Weekly Report. 1983;32(32):417-8.

46. Plague Pneumonia — California. Morbidity and Mortality Weekly Report. 1984;33(34):481-3.

47. Winter plague--Colorado, Washington, Texas, 1983-1984. MMWR Morb Mortal Wkly Rep. 1984;33(11):145-8. Epub 1984/03/23. PubMed PMID: 6422229.

48. Weniger BG, Warren AJ, Forseth V, Shipps GW, Creelman T, Gorton J, et al. Human bubonic plague transmitted by a domestic cat scratch. Jama. 1984;251(7):927-8. Epub 1984/02/17. PubMed PMID: 6694293.

49. Welty TK, Grabman J, Kompare E, Wood G, Welty E, Van Duzen J, et al. Nineteen cases of plague in Arizona. A spectrum including ecthyma gangrenosum due to plague and plague in pregnancy. West J Med. 1985;142(5):641-6. Epub 1985/05/01. PubMed PMID: 4013279; PubMed Central PMCID: PMCPMC1306131.

50. Florman AL, Spencer RR, Sheward S. Multiple lung cavities in a 12-year-old girl with bubonic plague, sepsis, and secondary pneumonia. Am J Med. 1986;80(6):1191-3. Epub 1986/06/01. doi: 10.1016/0002-9343(86)90684-4. PubMed PMID: 3728514.

51. Wong TW. Plague in a pregnant patient. Trop Doct. 1986;16(4):187-9. Epub 1986/10/01. doi: 10.1177/004947558601600418. PubMed PMID: 3775853.

52. Moreno AJ, Reeves TA, Rodriguez AA, Turnbull GL. Imaging in plague. Eur J Nucl Med. 1987;13(6):315-7. Epub 1987/01/01. doi: 10.1007/bf00256560. PubMed PMID: 3665983.

53. Human Plague — United States, 1988. Morbidity and Mortality Weekly Report. 1988;37(42):653-6.

54. Imported bubonic plague--District of Columbia. MMWR Morb Mortal Wkly Rep. 1990;39(49):895, 901. Epub 1990/12/14. PubMed PMID: 2123285.

55. Migden D. Bubonic plague in a child presenting with fever and altered mental status. Ann Emerg Med. 1990;19(2):207-9. Epub 1990/02/01. doi: 10.1016/s0196-0644(05)81811-6. PubMed PMID: 2301801.

56. Owens C. Plague in New Mexico. Journal of Community Health Nursing. 1990;7(3):153-8.

57. McClean KL. An outbreak of plague in northwestern province, Zambia. Clin Infect Dis. 1995;21(3):650-2. Epub 1995/09/01. doi: 10.1093/clinids/21.3.650. PubMed PMID: 8527559.

58. Fatal human plague--Arizona and Colorado, 1996. MMWR Morb Mortal Wkly Rep. 1997;46(27):617-20. Epub 1997/07/11. PubMed PMID: 9218646.

59. Gage KL, Dennis DT, Orloski KA, Ettestad P, Brown TL, Reynolds PJ, et al. Cases of cat-associated human plague in the Western US, 1977-1998. Clin Infect Dis. 2000;30(6):893-900. Epub 2000/06/15. doi: 10.1086/313804. PubMed PMID: 10852811.

60. Imported plague--New York City, 2002. MMWR Morb Mortal Wkly Rep. 2003;52(31):725-8. Epub 2003/08/09. PubMed PMID: 12904738.

61. Chmura K, Cool C, Kircher T, Chan ED. Painful lymphadenopathy and fulminant sepsis in a previously healthy 16-year-old girl. Chest. 2003;124(1):379-82. Epub 2003/07/11. doi: 10.1378/chest.124.1.379. PubMed PMID: 12853549.

62. Human plague--four states, 2006. MMWR Morb Mortal Wkly Rep. 2006;55(34):940-3. Epub 2006/09/01. PubMed PMID: 16943764.

63. Notes from the field: two cases of human plague--Oregon, 2010. MMWR Morb Mortal Wkly Rep. 2011;60(7):214. Epub 2011/02/25. PubMed PMID: 21346709.

64. Tourdjman M, Ibraheem M, Brett M, Debess E, Progulske B, Ettestad P, et al. Misidentification of Yersinia pestis by automated systems, resulting in delayed diagnoses of human plague infections--Oregon and New Mexico, 2010-2011. Clin Infect Dis. 2012;55(7):e58-60. Epub 2012/06/21. doi: 10.1093/cid/cis578. PubMed PMID: 22715170.

65. Lazet K. Case report. The Journal of Family Practice. 2018;67:3.

66. Melman SD, Ettestad PE, VinHatton ES, Ragsdale JM, Takacs N, Onischuk LM, et al. Human case of bubonic plague resulting from the bite of a wild Gunnison's prairie dog during translocation from a plague-endemic area. Zoonoses Public Health. 2018;65(1):e254-e8. Epub 2017/11/08. doi: 10.1111/zph.12419. PubMed PMID: 29110441; PubMed Central PMCID: PMCPMC5859330.

67. Kehrmann J, Popp W, Delgermaa B, Otgonbayar D, Gantumur T, Buer J, et al. Two fatal cases of plague after consumption of raw marmot organs. Emerg Microbes Infect. 2020;9(1):1878-80. Epub 2020/08/09. doi: 10.1080/22221751.2020.1807412. PubMed PMID: 32762515; PubMed Central PMCID: PMCPMC7473306.

68. Dale AP, Kretschmer M, Ruberto I, Wagner DM, Solomon C, Komatsu K, et al. Notes from the Field: Delays in Identification and Treatment of a Case of Septicemic Plague - Navajo County, Arizona, 2020. MMWR Morb Mortal Wkly Rep. 2021;70(31):1063-4. Epub 2021/08/06. doi: 10.15585/mmwr.mm7031a1. PubMed PMID: 34351879; PubMed Central PMCID: PMCPMC8367317 Journal Editors form for disclosure of potential conflicts of interest. No potential conflicts of interest were disclosed.

69. Haddad C, Valero A. Streptomycin in bubonic plague. Br Med J. 1948;1(4560):1026. Epub 1948/05/29. doi: 10.1136/bmj.1.4560.1026. PubMed PMID: 18860439; PubMed Central PMCID: PMCPMC2090756.

70. Nguyen Van A, Nguyen Duc H, Pham Van D, Nguyen Van L. Letter: Co-trimoxazole in bubonic plague. Br Med J. 1973;4(5884):108-9. Epub 1973/10/13. doi: 10.1136/bmj.4.5884.108-c. PubMed PMID: 4745331; PubMed Central PMCID: PMCPMC1587212.

71. Butler T, Levin J, Linh NN, Chau DM, Adickman M, Arnold K. Yersinia pestis infection in Vietnam. II. Quantiative blood cultures and detection of endotoxin in the cerebrospinal fluid of patients with meningitis. J Infect Dis. 1976;133(5):493-9. Epub 1976/05/01. doi: 10.1093/infdis/133.5.493. PubMed PMID: 1262715.

72. Palmer DL, Kisch AL, Williams RC, Reed WP. Clinical Features of Plague in the United States: The 1969-1970 Epidemic. The Journal of Infectious Diseases. 1971;124(4):367-71.

73. Butler T, Bell WR, Nguyen Ngoc L, Nguyen Dinh T, Arnold K. Yersinia pestis infection in Vietnam. I. Clinical and hematologic aspects. J Infect Dis. 1974;129:Suppl:S78-84. Epub 1974/05/01. doi: 10.1093/infdis/129.supplement_1.s78. PubMed PMID: 4825250.

74. von Reyn CF, Weber NS, Tempest B, Barnes AM, Poland JD, Boyce JM, et al. Epidemiologic and clinical features of an outbreak of bubonic plague in New Mexico. J Infect Dis. 1977;136(4):489-94. Epub 1977/10/01. doi: 10.1093/infdis/136.4.489. PubMed PMID: 908848.

75. Barnes AM, Poland JD. Plague in the United States, 1982. Morbidity and Mortality Weekly Report: Surveillance Summaries. 1983;32(3SS):19SS-24SS.

76. Becker TM, Poland JD, Quan TJ, White ME, Mann JM, Barnes AM. Plague meningitis--a retrospective analysis of cases reported in the United States, 1970-1979. West J Med. 1987;147(5):554-7. Epub 1987/11/01. PubMed PMID: 3424819; PubMed Central PMCID: PMCPMC1025943.

77. Crook LD, Tempest B. Plague. A clinical review of 27 cases. Arch Intern Med. 1992;152(6):1253-6. Epub 1992/06/01. doi: 10.1001/archinte.152.6.1253. PubMed PMID: 1599354.

78. Chanteau S, Ratsitorahina M, Rahalison L, Rasoamanana B, Chan F, Boisier P, et al. Current epidemiology of human plague in Madagascar. Microbes Infect. 2000;2(1):25-31. Epub 2000/03/16. doi: 10.1016/s1286-4579(00)00289-6. PubMed PMID: 10717537.

79. Boulanger LL, Ettestad P, Fogarty JD, Dennis DT, Romig D, Mertz G. Gentamicin and tetracyclines for the treatment of human plague: review of 75 cases in new Mexico, 1985-1999. Clin Infect Dis. 2004;38(5):663-9. Epub 2004/02/27. doi: 10.1086/381545. PubMed PMID: 14986250.

80. Cabanel N, Leclercq A, Chenal-Francisque V, Annajar B, Rajerison M, Bekkhoucha S, et al. Plague outbreak in Libya, 2009, unrelated to plague in Algeria. Emerg Infect Dis. 2013;19(2):230-6. Epub 2013/01/26. doi: 10.3201/eid1902.121031. PubMed PMID: 23347743; PubMed Central PMCID: PMCPMC3559055.

81. Sinyange N, Kumar R, Inambao A, Moonde L, Chama J, Banda M, et al. Outbreak of Plague in a High Malaria Endemic Region - Nyimba District, Zambia, March-May 2015. MMWR Morb Mortal Wkly Rep. 2016;65(31):807-11. Epub 2016/08/12. doi: 10.15585/mmwr.mm6531a4. PubMed PMID: 27513350.

82. Apangu T, Griffith K, Abaru J, Candini G, Apio H, Okoth F, et al. Successful Treatment of Human Plague with Oral Ciprofloxacin. Emerg Infect Dis. 2017;23(3):553-5. Epub 2017/01/27. doi: 10.3201/eid2303.161212. PubMed PMID: 28125398; PubMed Central PMCID: PMCPMC5382724.

83. Mwengee W, Butler T, Mgema S, Mhina G, Almasi Y, Bradley C, et al. Treatment of plague with gentamicin or doxycycline in a randomized clinical trial in Tanzania. Clin Infect Dis. 2006;42(5):614-21. Epub 2006/02/01. doi: 10.1086/500137. PubMed PMID: 16447105.
