# Supplementary material for: A systematic review of the clinical profile of patients with bubonic plague and the outcome measures used in research settings
Source: PLoS Negl Trop Dis. 2023 Nov 9;17(11):e0011509. doi: 10.1371/journal.pntd.0011509 (PMC10662759; doi:10.1371/journal.pntd.0011509)
Supplement: Attachment — Submitted filename: Response to reviewers_14Sep2023.docx [file pntd.0011509.s007.docx]

Dear Reviewers,

Thank you for your feedback on our article titled “A systematic review of the clinical profile of patients with bubonic plague and the outcome measures used in research settings”

Please find below a table containing our point-by-point response to your comments.

Sincerely,

Josephine Bourner

| **Feedback** | **Response** |
| --- | --- |
| Table 3: Presentation of the results is not concordant with the number of reported outcomes. The authors should choose between to present the number of death and full recovery among the 1300 reported outcomes and to present the distribution of the outcomes among the 1343 patients and in this case, precise the number of unknown outcomes | For the total patient population included in this systematic review, we have revised the table to present the n/N (%) of:   - Deaths - Full recoveries - Unknown outcomes |
| Table 3: It will be worthy to indicate in table 3, the % of deaths observed in high-income countries and in low-and middle-income countries and the sex ratio of deaths in these 2 categories | We have reconfigured Table 3 to present the data for:   - All patients - Patients in HICs - Patients in LMIC   We did not extract outcome data according to sex, so this information is not presented. |
| Table 3: Persistent bubos already are mentioned in table 2 and should be removed from table 3 | N (%) of persistent buboes has been removed from Table 3. |
| There is a discordance in the number of patients with information on intervention. Lines 266-268: information was available for 305 patients, but 271 +24 +11 =306. The same discordance is present also in table 4. | This error has now been identified and we confirm the correct denominator is 306. This has been updated in the text and in Table 4. |
| It would be easier for the reader if the authors provided figures that supported the conclusions that could be drawn from the very large tables. For example, the authors could display graphically the most common outcomes reported; the authors could display in a graph, a comparison between the outcomes in the US compared to Madagascar, since these were the two largest groups; the authors could compare outcomes over time in all groups using a graph. | Having drafted the suggested figures, we feel they are potentially misleading:   - Presenting CFR (and other outcomes over time) graphically offers little insight in to trends in outcomes over time. CFR in both HIC and LMICs appears to worsen from the early 1900s to 2021. However, rather than worsening patient outcomes, this more likely represents improved data collection in larger clinical studies - Equally the comparison of outcomes in the US and outcomes in Madagascar is uninformative as most of the US data derive from multiple case reports from the 1960s to the 1990s, while the Madagascar data derives from a single cohort study in the late 1990s. There are inherent differences in the case selection in these studies and standard of care available in their respective countries during the different time periods, the outcomes between the two countries are therefore not comparable. |
| The authors provide a somewhat limited discussion of how these data can be helpful to advance the impact of clinical trials for plague | We believe we have adequately demonstrated the impact of our results on the definition of primary endpoints in future trials for bubonic plague. |
| Line 49: pestis should be written without upper-case letter | This typing error has been corrected. |
| Line 225: range of temperature: 36°C to 103.2°C. Is it possible? or is it a problem of unit? | This was a problem of unit. I have converted 103.2°F to 39.5°F and updated the upper limit of the range accordingly. Note: the median did not change with this correction. |
| Table 1: References are missing for 5 studies:   - Three cases of bubonic plague arising in England, 1916 - Plague : Shasta County, California, 1965 - Suspected Case of Imported bubonic plague, 1966 - Bubonic Plague – California, 1970 - Case report, Lazet, 2018 | The reference list has been checked and all missing references in Table 1 have been added. |
| While providing valuable data for understanding the clinical feature of bubonic plague, this review also presents a limitation, which should be discussed in this manuscript. The data collection was only limited in papers in English. There are many reports on bubonic plague around the world in other languages, such as reports in Russian and Chinese. Much more patients have been missed from this statistical analysis. When discuss the limitation, Xu’s paper needs to be cited (Xu, et al. Nonlinear effect of climate on plague during the third pandemic in China. PNAS 2011 June 6.).  The flowing review is also recommended to cite in the section of introduction. Yang et al. Zoonoses (2023) 3:5; DOI 10.15212/ZOONOSES-2022-0040 It is recommended that the big Table 1 should be moved to supplements. | We have included in the discussion an acknowledgement that only studies written in English are included in this review, and cited Xu et al.  We have not removed Table 1 to a supplementary file as we believe the reader will need to see the included papers and their characteristics in the paper as opposed to having to consult supplementary files. However, we will leave it for the Editor to decide. |
